# Bake your phantom—low-cost recipes for dough-based, tissue-mimicking CT phantoms

**DOI:** 10.1007/s00330-025-11887-5

**Published:** 2025-08-04

**Authors:** Sonja Wichelmann, Florian Weiler, Thomas Friedrich, Joerg Barkhausen, Roman Kloeckner, Franz Wegner, Malte Maria Sieren

**Affiliations:** 1 Fraunhofer Institute for Digital Medicine MEVIS, Lübeck, Germany; 2 Fraunhofer Institute for Digital Medicine MEVIS, Bremen, Germany; 3 Fraunhofer Research Institution for Individualized and Cell-Based Medical Engineering IMTE, Lübeck, Germany; 4 Institute of Radiology and Nuclear Medicine, University Hospital Schleswig-Holstein, Lübeck, Germany; 5 Institute of Interventional Radiology, University Hospital Schleswig-Holstein, Lübeck, Germany

**Keywords:** Phantoms (Imaging), Tomography (X-ray computed), Liver, Models (anatomic), Printing (3D)

## Abstract

**Objective:**

Phantoms are essential for minimizing radiation-intensive experiments on humans and enhancing patient safety through model-based teaching and experimentation. While many phantoms are commercially available, their widespread use is limited by manufacturing complexity and high costs. This study aims to demonstrate a dough-based method to create customizable, realistic CT phantoms using affordable, readily available ingredients.

**Materials and methods:**

For this study, various doughs composed of flour, salt, water, and oil were created, scanned, and evaluated to assess their suitability for CT applications. Additionally, the effects of storage conditions, preservation, and temperature variations were analyzed. As an example, a liver was segmented from a 3D CT scan, scaled to 1:2, and a negative mold was 3D printed. The mold was subsequently filled with the most suitable dough composition to replicate the organ’s anatomy and density.

**Results:**

The evaluation of the scanned ingredients and doughs demonstrated that Hounsfield unit (HU) values ranging from below −200 HU to above 1200 HU can be achieved, enabling the simulation of various human tissue densities. Based on the analysis, simple recipes are proposed to replicate radiodensities of different anatomical structures. Additionally, the results from the liver phantom confirm the feasibility of mimicking liver tissue and morphology.

**Conclusions:**

CT phantoms with specific radiodensities, mimicking human tissues, can be created using simple dough recipes, as demonstrated with our liver CT phantom. Refrigeration or freezing extends usability for a longer time, but temperature effects must be considered to ensure accurate HU values in CT scans.

**Key Points:**

***Question*** *CT phantoms are costly and complex, limiting accessibility for customized imaging studies. Can common ingredients from the kitchen be used to create tissue-mimicking phantoms*?

***Findings*** *The dough-based phantoms simulated radiodensities from −200 to 1200+ HU, enabling the simulation of various human tissue densities*.

***Clinical relevance*** *Affordable, anatomically accurate CT phantoms can be made from kitchen ingredients for training and research, as demonstrated with our liver CT phantom*.

**Graphical Abstract:**

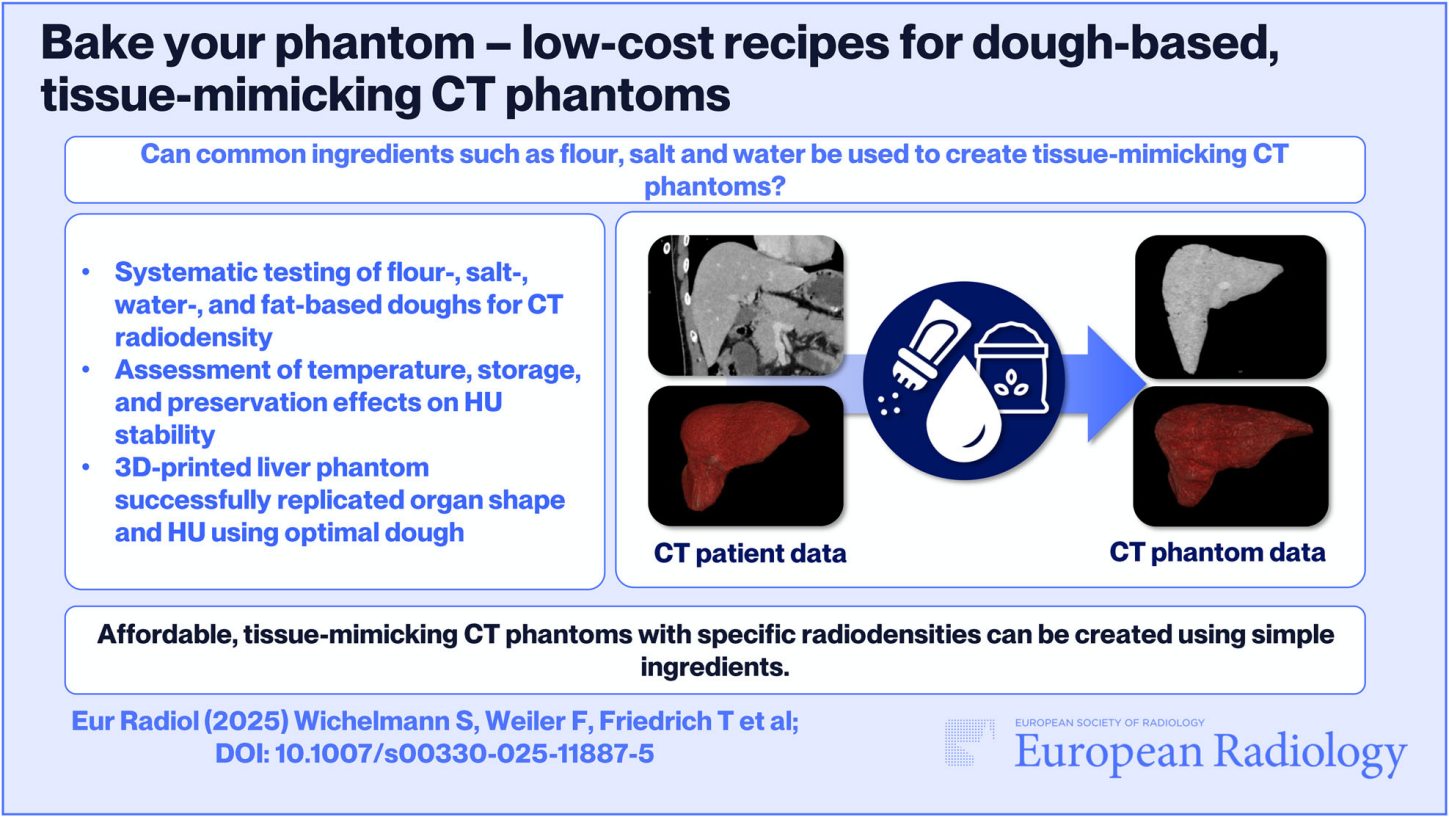

## Introduction

Human tissue-mimicking CT phantoms have numerous applications, including use in experimental research and test data generation [1, 2]. Additional applications involve imaging system calibration, quality control, and educational purposes [3]. While specific requirements for phantoms vary, key features are a realistic, anthropomorphic anatomy and Hounsfield density in CT scans.

Currently, commercially available, ready-to-use CT phantoms are offered by companies such as Erler-Zimmer GmbH & Co.KG and True Phantom Solutions Inc. These phantoms accurately replicate various body parts and are valuable for scientific and educational applications [4]. Skull phantoms, for instance, have been utilized in segmentation algorithm evaluations [5], reference value comparisons [6], and brain shift compensation studies in CBCT [7]. Although these phantoms often provide highly realistic representations, their high production cost, limited availability, and lack of options for researchers to produce or modify them independently present significant limitations.

Various techniques exist for fabricating CT phantoms that mimic human tissue [8]. Many of these methods rely on 3D printing and synthetic materials [9]. For example, synthetic polymers such as polymethyl methacrylate and silicone are commonly used to simulate soft tissue [10–12], while gypsum is employed for bone replication [13]. However, various materials with different properties are needed to simulate the radiodensity of human tissue, expensive equipment and materials are required, and the phantom printing can be time-consuming.

This work introduces an unconventional, yet simple, and low-cost method for fabricating customizable CT phantoms using readily available materials. We propose the development of dough-based phantoms created from common ingredients such as flour, salt, fat, and water. By adjusting ingredient ratios, different doughs were produced. These were then scanned, and analyzed to determine their radiodensity in CT images. Importantly, the resulting dough exhibited sufficient consistency to maintain shape stability, enabling the creation of phantoms that accurately replicate anatomical forms. In this study, we suggest recipes based on affordable, readily available ingredients for CT phantoms and show the feasibility with the creation of a liver phantom.

## Materials and methods

### Phantom preparation

Ethical approval was not required for this study, as no direct participation of human volunteers or patients was necessary (waiver granted by the local ethics committee).

#### Ingredients

The following ingredients were used: flour (wheat flour type 405, Diamant), salt (iodized salt with fluoride, Gut & Günstig), margarine based on rapeseed, coconut and palm oil with 80% fat in total and 20% water (Alsan-S plant margarine, Alsan), plant fat based on palm, coconut and sunflower oil with 100% fat in total (Biskin) and citric acid (Heitmann).

#### Dough compositions

The simplest method for creating a basic dough is the combination of water and flour, and salt can be added for modeling purposes. Salt, due to its elevated density and increased X-ray absorption, results in a higher Hounsfield Unit (HU) value. To investigate this effect, flour and salt were mixed in varying ratios, followed by the addition of water to achieve a kneadable consistency. A standardized sample weight of 40 g of dry ingredients was selected to ensure uniformity across experiments, allowing the material to fit precisely within the compartments of the ice cube mold. Doughs with salt concentrations of *x* = {10%, 20%, …, 90%, 100%} by weight were prepared.

To explore the influence of lower HU materials, additional dough compositions were formulated using fat in place of water, as fat typically exhibits lower HU values. For this variation, 40 g of flour was combined with plant-based fat or margarine. The quantities of water or fat to achieve the desired consistency were carefully documented. Considering the necessity for repeated use of dough-based phantoms over extended periods, various storage and preservation methods were evaluated. One preservation approach involved the incorporation of preservative ingredients. In addition to salt, citric acid was selected for its known preservation properties. A dough containing 90% flour and 10% citric acid by weight was prepared, with water added to achieve the desired consistency. This citric acid concentration was chosen to balance preservation efficacy while mitigating potential alterations to the dough’s structural integrity.

All ingredients were used at room temperature. For the creation of each dough sample, a clean bowl and spoon were used to avoid contamination by previously created doughs. The ingredients were carefully mixed by hand using a spoon until a homogenous structure was achieved. Each dough and ingredient sample was wrapped in plastic film following preparation and subsequently placed into an ice cube mold featuring 15 compartments arranged in three rows and five columns. Each compartment measured 30 × 30 × 27 mm.

#### Effect of reconstruction kernel on HU measurements

To assess the influence of different CT reconstruction kernels on HU values, one representative dough sample (40 g flour + 25 g margarine) was reconstructed using four commonly applied clinical kernels: B20f, B31f, B50f, and B80f. These kernels vary in sharpness and noise characteristics.

#### Anthropomorphic liver phantom example

To exemplify the methodology, a liver phantom was fabricated utilizing a 3D-printed mold derived from a patient CT scan (Ethics reference number: 2023-196 1 15.10.2024/IK). The liver segmentation process employed a convolutional neural network-based deep learning algorithm, optimized for abdominal CT scans [14]. The segmented liver model was refined through Laplacian surface smoothing to mitigate voxel artifacts.

A hollow mold of the liver was created by subtracting the liver shape from a scaled duplicate, resulting in a wall thickness of 1.5 mm. This mold was bisected to facilitate dough filling. The mold was fabricated at 50% of the liver’s original size using a polyjet 3D printer (J850 Prime from Stratasys) in high-mix mode with a layer resolution of 27 µm. To achieve precise anatomical representation while maintaining durability, the mold material consisted of a composite blend of elastic Agilus30clear and rigid VeroBlackPlus (Digital Material RGDA8455-DM).

Healthy liver tissue typically exhibits radiodensity values ranging between 48.9 HU and 78.2 HU [15], whereas fatty liver tissue has lower HU values. Accordingly, the dough with HU values within this range was selected. The liver mold was filled with the dough by compressing it into each half, ensuring complete filling before assembly.

### Evaluation

All phantom samples and ingredients underwent CT imaging using a Siemens SOMATOM Definition AS+ clinical CT scanner. Those scans were performed with a voxel size of 0.30–0.44 × 0.30–0.44 × 1 mm and convolution kernel B20f. For each scan, one single large cubic region of interest was manually delineated, and larger air gaps or mold imperfections were excluded. The median HU ${Q}_{50 \% }$, as well as the 25% and 75% quantiles $[{Q}_{25 \% },\,{Q}_{75 \% }]$, were calculated for each region of interest to assess HU variability. The liver phantom was scanned with a voxel size of 0.45 × 0.45 × 0.9 mm using a convolution kernel B31f.

The samples were subjected to CT scanning immediately following preparation at room temperature to establish baseline measurements. Subsequently, the samples were stored in a refrigerator at 6 °C for a duration of 24 h, after which they underwent rescanning to assess potential radiodensity changes. Then, the samples were relocated to a freezer maintained at −18 °C for an additional 24-h period before being subjected to a final CT scan. Temperature fluctuations are known to modulate the density of materials, thereby affecting the corresponding HU values, which are critical for CT-based analyses [16].

## Results

### General phantom properties

The resulting doughs produced phantoms of varying volumes, influenced by the salt content. As salt has a higher density than flour, increasing the salt concentration led to a reduction in dough volume, resulting in less surplus material when filling the ice cube mold. A notable decrease in flexibility was observed with higher salt concentrations. Doughs containing 80% or more salt were prone to crumbling, making it challenging to form cohesive structures. Encasing the dough in plastic film during shaping helped maintain structural integrity.

Table 1 documents the quantities of flour, salt, citric acid, water, margarine, and plant fat required for each dough composition. The amount of water required inversely correlated with salt concentration—doughs with higher salt content required less water. Conversely, fat-based doughs required larger amounts of fat compared to water-based ones.

**Table 1:** Measured weights of all created doughs

Dough mixture	Flour	Salt	Citric acid	Water	Margarine	Plant fat
100% salt + water	0.0 g	40.0 g	0.0 g	6.0 g	0.0 g	0.0 g
90% salt, 10% flour + water	4.0 g	36.0 g	0.0 g	7.6 g	0.0 g	0.0 g
80% salt, 20% flour + water	8.0 g	32.0 g	0.0 g	8.2 g	0.0 g	0.0 g
70% salt, 30% flour + water	12.0 g	28.0 g	0.0 g	9.4 g	0.0 g	0.0 g
60% salt, 40% flour + water	16.0 g	24.0 g	0.0 g	10.8 g	0.0 g	0.0 g
50% salt, 50% flour + water	20.0 g	20.0 g	0.0 g	13.0 g	0.0 g	0.0 g
40% salt, 60% flour + water	24.0 g	16.0 g	0.0 g	14.8 g	0.0 g	0.0 g
30% salt, 70% flour + water	28.0 g	12.0 g	0.0 g	16.6 g	0.0 g	0.0 g
20% salt, 80% flour + water	32.0 g	8.0 g	0.0 g	18.2 g	0.0 g	0.0 g
10% salt, 90% flour + water	36.0 g	4.0 g	0.0 g	20.6 g	0.0 g	0.0 g
90% flour, 10% citric acid + water	36.0 g	0.0 g	4.0 g	20.6 g	0.0 g	0.0 g
Flour + margarine	40.0 g	0.0 g	0.0 g	0.0 g	25.0 g	0.0 g
Flour + plant fat	40.0 g	0.0 g	0.0 g	0.0 g	0.0 g	40.0 g

### Computed tomography properties

**Fig. 1:**
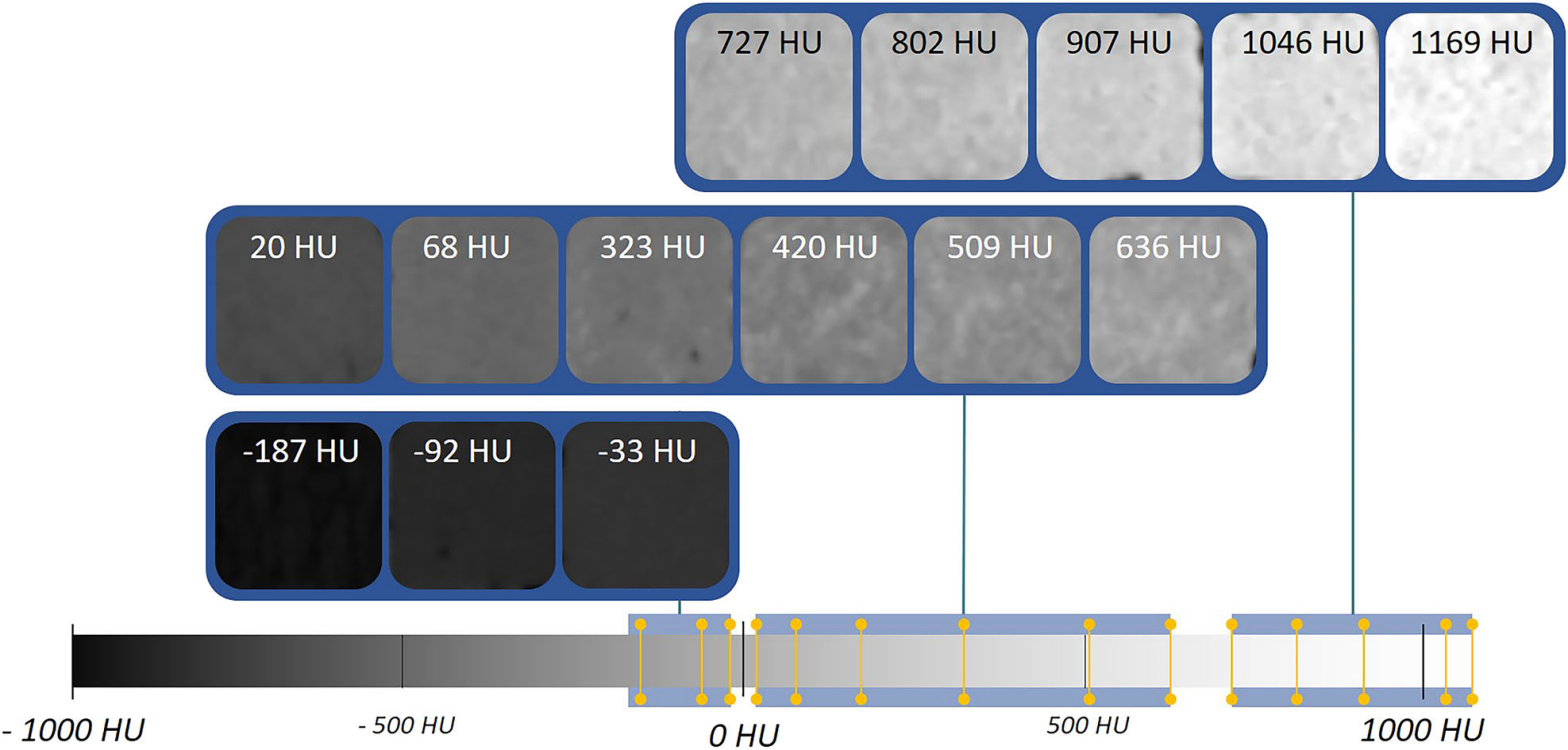
Examples of various samples with their corresponding density values. At the bottom of the image, there is a Hounsfield scale with highlighted regions (blue background) and the exact mean values (yellow marks) of the samples shown here. Additional samples with matching formulations and density values can be found in Tables 1 and 2

**Fig. 2:**
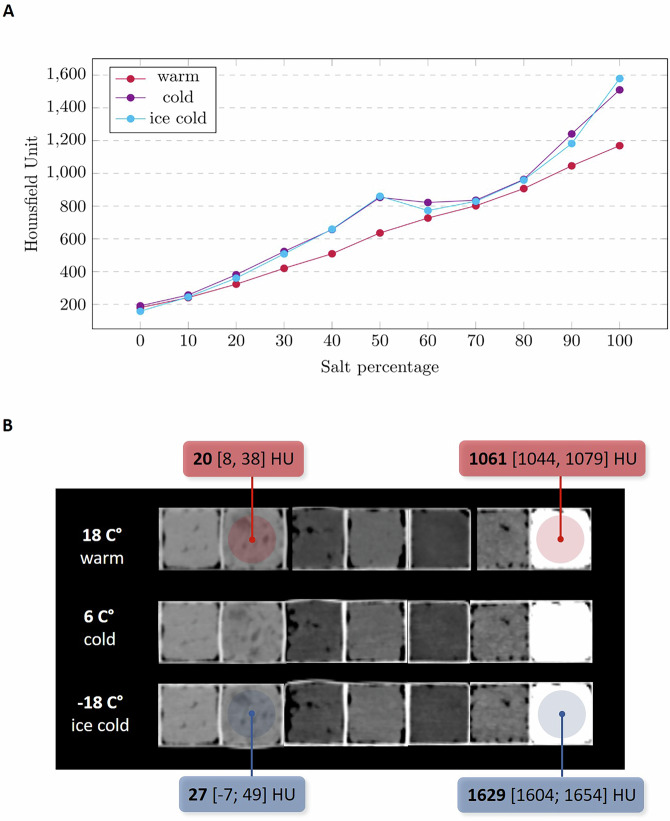
**A** The resulting median intensities of doughs prepared with mixtures containing *x*% salt and 100% − *x*% flour (including 10% citric acid for the 0% salt formulation) are shown for warm (18 °C, red), cold (6 °C, purple), and ice-cold (−18 °C, cyan) temperatures. **B** Sample densities of the margarine-based dough, the plant fat-based dough, plant fat, margarine, flour, citric acid, and salt were measured at different temperatures. Two sample pairs are presented, highlighting representative density values

Figure 1 shows CT reconstructions of different samples, their placement on the Hounsfield scale, and corresponding images of analogous CT tissues. Figure 2 presents the median HU values of the salty doughs depending on the salt percentage and sample scans at various temperatures.

Doughs with minimal or no salt exhibited air inclusions, whereas doughs with high salt concentrations displayed more homogenous structures. Plant fat-based doughs revealed minor fat globules. A clear positive correlation between salt concentration and radiodensity was observed at room temperature, with continuous increases in HU values. However, at lower temperatures, the HU values plateaued or slightly decreased for doughs containing 60% to 80% salt.

### HU of phantoms and ingredients

Table 2 presents the median, 25%, and 75% quantile HU values for all scanned objects. Phantoms with high salt concentrations exhibited a non-linear increase in HU values, while salt-free doughs demonstrated lower HU values. Among the doughs, plant fat-based ones exhibited the lowest HU values. Negative HU values were recorded for several ingredients, including flour and plant fat, followed by margarine and citric acid. The HU variability for most materials ranged between 10 HU and 40 HU from the median. Doughs with 50% to 100% salt content displayed higher deviations, particularly at colder temperatures. Using different reconstruction kernels produced mean HU values of 63 ± 5 HU (B20f), 61 ± 5 HU (B31f), 71 ± 14 HU (B50f), and 74 ± 16 HU (B80f).

**Table 2:** Resulting HU values of the created doughs and ingredients for the different temperatures

Dough mixture/ingredients	Warm (18 °C): ${{\mathbf{Q}}}_{50 \% },$ ${{\mathbf{Q}}}_{25 \% }-{Q}_{75 \% }$ HU	Cold (6 °C): ${{\mathbf{Q}}}_{50 \% },$ ${{\mathbf{Q}}}_{25 \% }-{Q}_{75 \% }$ HU	Ice cold (−18 °C): ${{\mathbf{Q}}}_{50 \% },\,{Q}_{25 \% }-{Q}_{75 \% }$ HU
100% salt + water	1169 [1098, 1225]	1510 [1404, 1608]	1579 [1427, 1680]
90% salt, 10% flour + water	1046 [989, 1093]	1241 [1155, 1341]	1182 [1098, 1269]
80% salt, 20% flour + water	907 [863, 941]	963 [837, 1130]	959 [848, 1116]
70% salt, 30% flour + water	802 [755, 840]	836 [761, 927]	829 [760, 896]
60% salt, 40% flour + water	727 [684, 768]	822 [744, 892]	773 [694, 842]
50% salt, 50% flour + water	636 [601, 671]	853 [795, 911]	860 [802, 917]
40% salt, 60% flour + water	509 [475, 544]	657 [602, 715]	659 [602, 717]
30% salt, 70% flour + water	420 [387, 454]	523 [464, 585]	508 [448, 572]
20% salt, 80% flour + water	323 [296, 343]	381 [324, 425]	360 [313, 402]
10% salt, 90% flour + water	241 [225, 256]	257 [222, 290]	245 [213, 276]
90% flour, 10% citric acid + water	180 [166, 193]	191 [172, 207]	158 [139, 175]
Flour + margarine	68 [58, 78]	71 [57, 85]	84 [70, 98]
Flour + plant fat	20 [8, 38]	19 [−7, 39]	27, [−7, 49]
Plant fat	−187 [−196, −177]	−194 [−209, 180]	−177 [−192, −164]
Margarine	−92 [−98, −85]	−88 [−101, 75]	−61 [−73, −49]
Flour	−207 [−223, −196]	−204 [−223, 189]	−202 [−219, −187]
Citric acid	−33 [−50, −17]	−44 [−67, −22]	−44, [−66, −23]
Salt	1061 [1044, 1079]	1616 [1591, 1642]	1629 [1604, 1654]

*HU* Hounsfield unit

### CT phantom recipes for human tissues

A combination of the HU values from various human tissues [17] with the ingredients quantities documented in Table 1 and the resulting HUs of the scanned doughs given in Table 2 allowed the development of recipes replicating different tissue types: flour closely matched the low HU values associated with skin tissue. Margarine and plant fat effectively simulated fat tissue. The flour-margarine and flour-plant-fat dough approximated muscle tissue HU values. Bone density was best replicated by various flour-salt dough compositions. To simulate the high HU values characteristic of enamel, high-salt phantoms scanned at cold temperatures were required. All recipes with the exact ingredient quantities, the resulting HUs depending on the temperature, and the human tissue to be simulated are shown in Table 3.

**Table 3:** HU values of different parts in the human body [17] and recipes for them

Human tissue	HU ranges	Median HU of recipe at 18/6/−18 °C	Recipe ingredients
Enamel	[1553, 2850]	-------/1616/1629	100% salt
Compact bone	[662, 1988]	1061/1616/1629	100% salt
		1169/1510/1579	40.0 g salt, 6.0 g water
		1046/1241/1182	36.0 g salt, 4.0 g flour, 7.6 g water
		907/963/959	32.0 g salt, 8.0 g flour, 8.2 g water
		802/836/829	28.0 g salt, 12.0 g flour, 9.4 g water
		727/822/773	24.0 g salt, 16.0 g flour, 10.8 g water
		-----/853/860	20.0 g salt, 20.0 g flour, 13.0 g water
Spongy bone	[148, 661]	636/-----/-----	20.0 g salt, 20.0 g flour, 13.0 g water
		509/657/659	16.0 g salt, 24.0 g flour, 14.8 g water
		420/523/508	12.0 g salt, 28.0 g flour, 16.6 g water
		323/381/360	8.0 g salt, 32.0 g flour, 18.2 g water
		241/257/245	4.0 g salt, 36.0 g flour, 20.6 g water
		180/191/158	4.0 g citric acid, 36.0 g flour, 20.6 g water
Muscle tissue	[−5, 135]	68/71/84	40 g flour, 25 g margarine
		20/19/27	40 g flour, 40 g plant fat
Fat tissue	[−205, −51]	−92/−88/−61	100% margarine
		−187/−194/−177	100% plant fat
Skin tissue	[−718, −177]	−187/−194/−177	100% plant fat
		−207/−204/−202	100% flour

*HU* Hounsfield unit

### Anthropomorphic liver phantom example

The dough, based on 40 g flour and 25 g margarine, yielded a median HU of 68. Thus, it is the most suitable dough recipe of those listed in Table 3 for simulating liver tissue, since healthy liver tissue has radiodensity values ranging between 48.9 HU and 78.2 HU [15]. For the liver phantom, 360 g of flour was mixed with 225 g of margarine, and the 3D-printed liver mold was carefully filled with the resulting dough. The mold had a total volume of approximately 545 mL.

**Fig. 3:**
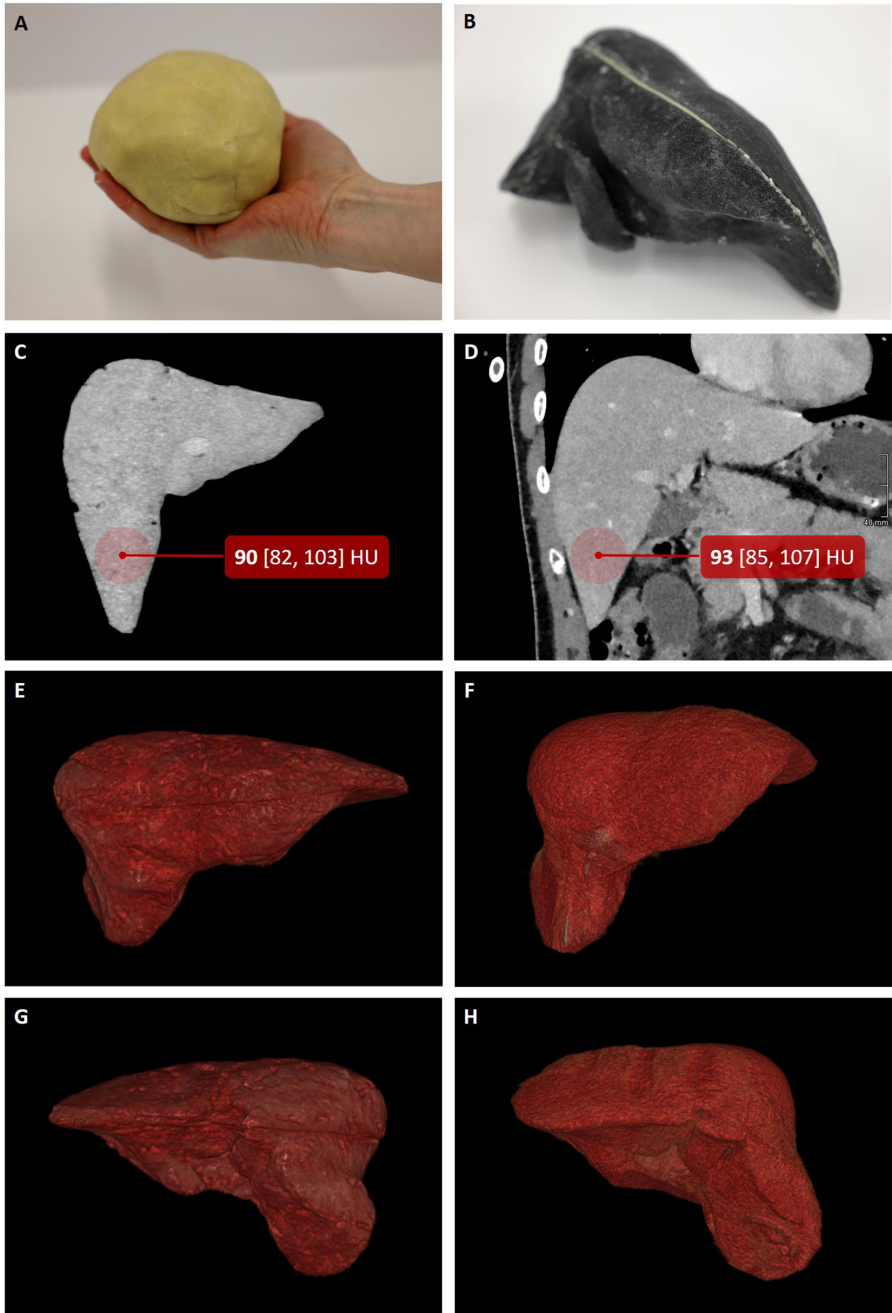
The margarine-based dough and the filled liver phantom are illustrated in the first row (**A**, **B**). The following images show a side-by-side comparison of the model (**C**, **E**, **G**) and the actual patient (**D**, **F**, **H**): the first row displays a coronal view of the CT scan, including an example of density measurement in the liver. The remaining images show 3D reconstructions of the front view (**C**, **D**) and rear view (**E**, **F**) of the phantom and the patient’s liver, based on CT data

Figure 3 displays a side-by-side comparison between the created liver phantom and the patient’s anatomy. The CT scan of the phantom revealed a largely homogenous internal structure, though minor air inclusions were present. The liver phantom exhibited a median HU value of 74, with a 25% quantile of 62 and a 75% quantile of 83, aligning closely with the HU range for healthy liver tissue.

## Discussion

This study systematically evaluated dough-based phantoms as an innovative, cost-efficient method for producing customizable CT phantoms. By examining various preservation techniques, storage conditions, and ingredient formulations, we derived recipes that mimic the radiodensities of distinct human tissues. The practical application of this methodology was demonstrated through the fabrication of a liver phantom using a 3D-printed mold, highlighting the versatility and feasibility of this approach.

This approach diverges from existing literature that predominantly focuses on synthetic materials and advanced 3D printing techniques [8]. Although simple recipes have been previously reported for other imaging modalities [18, 19], to our knowledge, this is the first documented use of common household ingredients such as flour, water, and fat to fabricate CT phantoms. In comparison to traditional CT phantom production, the proposed method offers notable advantages in simplicity, accessibility, and cost-effectiveness. The required materials are readily available, affordable, and can be mixed in minutes, enabling rapid prototyping without the need for sophisticated laboratory equipment. Furthermore, the phantoms can be manually shaped or cast into 3D-printed molds to replicate complex anatomical structures with relative ease. Notably, the constituent ingredients consistently displayed lower HU values compared to their dough compositions. For example, flour alone produced lower HU values relative to flour-water dough, suggesting that the hydration and subsequent aggregation of flour particles increased overall density. It was found that temperature was a significant modulator of radiodensity, with salt-rich doughs exhibiting pronounced increases in HU values at reduced temperatures. Measurements revealed HU elevations of up to 600 at lower temperatures, underscoring the propensity for salt to densify under cold conditions. This behavior likely reflects the crystallization dynamics of salt, which increases radiodensity and impacts the overall properties of the phantom. The variability observed in HU values among highly saline doughs may be attributed to CT artifacts stemming from high-density inclusions. Margarine-based phantoms exhibited comparatively modest HU shifts (approximately 30 HU) across temperature ranges, while flour-based doughs demonstrated only minimal temperature sensitivity. The results emphasize the necessity for comprehensive ingredient integration to ensure homogenous structures, as evidenced by the heterogeneity observed in plant fat-based doughs. Regarding acquisition parameters, sharper kernels such as B50f and B80f produced slightly higher HU values with increased standard deviations, reflecting elevated image noise. In contrast, smoother kernels like B20f and B31f yielded more consistent, lower-noise measurements. These findings indicate that reconstruction kernel choice can affect both the absolute HU value and its variability, and should be taken into account when reproducing phantom-based tissue simulations or comparing results across institutions. If a specific HU value is to be simulated, the most suitable recipe can be selected from Table 3. When the exact resulting HU value of the phantom is not that important, we recommend choosing a recipe with less salt and more flour. This is because more salt results in more crumbling of the dough and makes the phantom creation harder. The liver phantom yielded a CT image that accurately approximated the organ’s anatomical contours and radiodensity. However, the presence of air inclusions within the model highlights the challenge of eliminating trapped air during the dough preparation and molding stages. Despite thorough compression and careful handling, small pockets of air remained, potentially influencing the homogeneity of the final phantom. However, these were primarily visible in the 1 mm slices and may not be relevant for most, especially training purposes. A key advantage of this method is its capacity for extended usage through refrigeration, freezing, or the incorporation of preservatives. The ingredients citric acid and salt have a preserving property and prevent mold growth. Furthermore, the storage in the refrigerator allows a phantom usage for several weeks, and in the freezer for even longer. This not only enhances the longevity of the phantoms but also provides a sustainable and biodegradable alternative to synthetic models. The ingredients are locally sourced, environmentally friendly, and simple to dispose of, contributing to the sustainability of this approach. Applications for such a phantom are manifold: Its customizable anatomy, low cost, and tunable radiodensity allow for realistic simulation of various tissues, which is especially relevant for CT-guided interventions. A particularly promising use case is CT-guided biopsy training. While the current study did not formally evaluate needle retention or target insertion, the material’s consistency and stability suggest it could accommodate biopsy needles and embedded lesion simulants (e.g., olives or raisins). This aligns with the growing interest in hands-on training tools for image-guided procedures. Several studies have demonstrated that phantoms can significantly improve training outcomes and procedural competence. For instance, Jahnke et al developed a 3D-printed radiopaque phantom for CT-guided procedures, rated highly realistic by interventional radiologists [20]. Similarly, Picard et al showed that incorporating phantom-based simulation into resident education enhanced confidence and technical proficiency in CT-guided biopsies [21]. Other works have validated phantoms as effective for needle placement exercises and for simulating lesion localization [22, 23]. As such, future work will assess the phantom’s usability for procedural training, including its capacity to withstand repeated needle insertions and simulate lesion detection. Beyond training, the phantom’s adjustable composition enables its use in CT imaging research. The broad HU range achievable with simple ingredient modifications supports its application in developing and validating image processing algorithms, such as segmentation, registration, and artifact correction. Previous studies have demonstrated the value of anatomically accurate phantoms in radiomics and image quality assessment. For example, Rinaldi et al introduced a heterogeneous lung phantom (“HeLLePhant”) to validate radiomic feature extraction from CT data [24], while Hatamikia et al used 3D-printed tumor phantoms to evaluate registration algorithms with realistic morphology and density variations [25]. Similarly, anthropomorphic phantoms have been critical for validating CT hardware and simulation environments. Shankar et al demonstrated how a physical chest phantom could be used alongside CT simulation software to confirm image fidelity and biomarker reproducibility [26], and Hoerner et al used 3D-printed models for accurate CT dosimetry studies, particularly in sensitive scenarios such as fetal exposure [27]. These applications suggest that our phantom concept may also serve as a low-cost, reproducible tool for imaging validation, particularly in early-stage development of new technologies.

Nevertheless, several limitations warrant consideration. The manual sculpting of dough is inherently constrained in its ability to replicate fine anatomical details, necessitating the use of 3D-printed molds for higher precision. Additionally, air pockets remain a significant challenge, as the kneading and molding process can inadvertently introduce voids, compromising the uniformity of the phantom. Care must be taken to minimize excessive stretching or compression, which can exacerbate air inclusions. Replicating tissues with complex or heterogeneous HU distributions poses further difficulties, as doughs generally produce uniform radiodensities. Accurate temperature control during CT scanning is essential to ensure reproducibility and precision, given the observed HU variability at different temperatures. Furthermore, a continued refinement of the recipes may yield more accurate replication of various human tissues, as it is given here more generally for bones and soft tissues.

## Conclusion

This study presents a novel, pragmatic approach to the fabrication of density-specific CT phantoms using readily available materials. The resulting phantoms successfully replicate the HU values of bone, muscle, fat, and soft tissue, with partial approximation of skin and enamel. By proper storage in the refrigerator or freezer, the created phantoms can be used for several weeks or months. However, the demonstrated temperature sensitivity highlights the necessity for meticulous temperature regulation during CT imaging to maintain consistent HU values. Future research may focus on refining formulations to mimic specific organs or tissues with greater fidelity, as exemplified by the liver phantom. Additionally, exploring the potential integration of dough-based materials in 3D printing could facilitate the production of more intricate and anatomically precise models. This advancement holds substantial promise for expanding access to affordable, customizable CT phantoms, promoting progress in medical imaging research, education, and training.

## Abbreviation

HU: Hounsfield unit(s)
